# The microbiome impacts host hybridization and speciation

**DOI:** 10.1371/journal.pbio.3001417

**Published:** 2021-10-26

**Authors:** Asia K. Miller, Camille S. Westlake, Karissa L. Cross, Brittany A. Leigh, Seth R. Bordenstein

**Affiliations:** 1 Vanderbilt University, Department of Biological Sciences, Nashville, Tennessee, United States of America; 2 Vanderbilt University, Vanderbilt Microbiome Innovation Center, Nashville, Tennessee, United States of America; 3 Vanderbilt University Medical Center, Vanderbilt Institute for Infection, Immunology and Inflammation, Nashville, Tennessee, United States of America; 4 Vanderbilt University Medical Center, Department of Pathology, Microbiology & Immunology, Nashville, Tennessee, United States of America

## Abstract

Microbial symbiosis and speciation profoundly shape the composition of life’s biodiversity. Despite the enormous contributions of these two fields to the foundations of modern biology, there is a vast and exciting frontier ahead for research, literature, and conferences to address the neglected prospects of merging their study. Here, we survey and synthesize exemplar cases of how endosymbionts and microbial communities affect animal hybridization and vice versa. We conclude that though the number of case studies remain nascent, the wide-ranging types of animals, microbes, and isolation barriers impacted by hybridization will likely prove general and a major new phase of study that includes the microbiome as part of the functional whole contributing to reproductive isolation. Though microorganisms were proposed to impact animal speciation a century ago, the weight of the evidence supporting this view has now reached a tipping point.

## Living and evolving in a microbial world

No macroorganism lives in isolation of the microbial world. Indeed, the human body houses roughly 37 trillion human and microbial cells (as well as viruses), the latter of which span bacteria, archaea, fungi, and some protists [1–7]. Across diverse hosts, microbiomes can range from simple to complex, labile to stable, and with high to weak transmission fidelity. The recognition of the widespread occurrence of host-associated microbiomes that consist of both obligate and nonobligate microbial associations has also spurred the growth and development of the structural terms holobiont and hologenome, which emphasize the multispecies and multigenomic nature of host–microbiome assemblage, respectively [8–13]. We use these structural definitions throughout this perspective without implication to any specific process or stability, as the nature of host–microbiome associations may vary across study systems.

**Fig 1:**
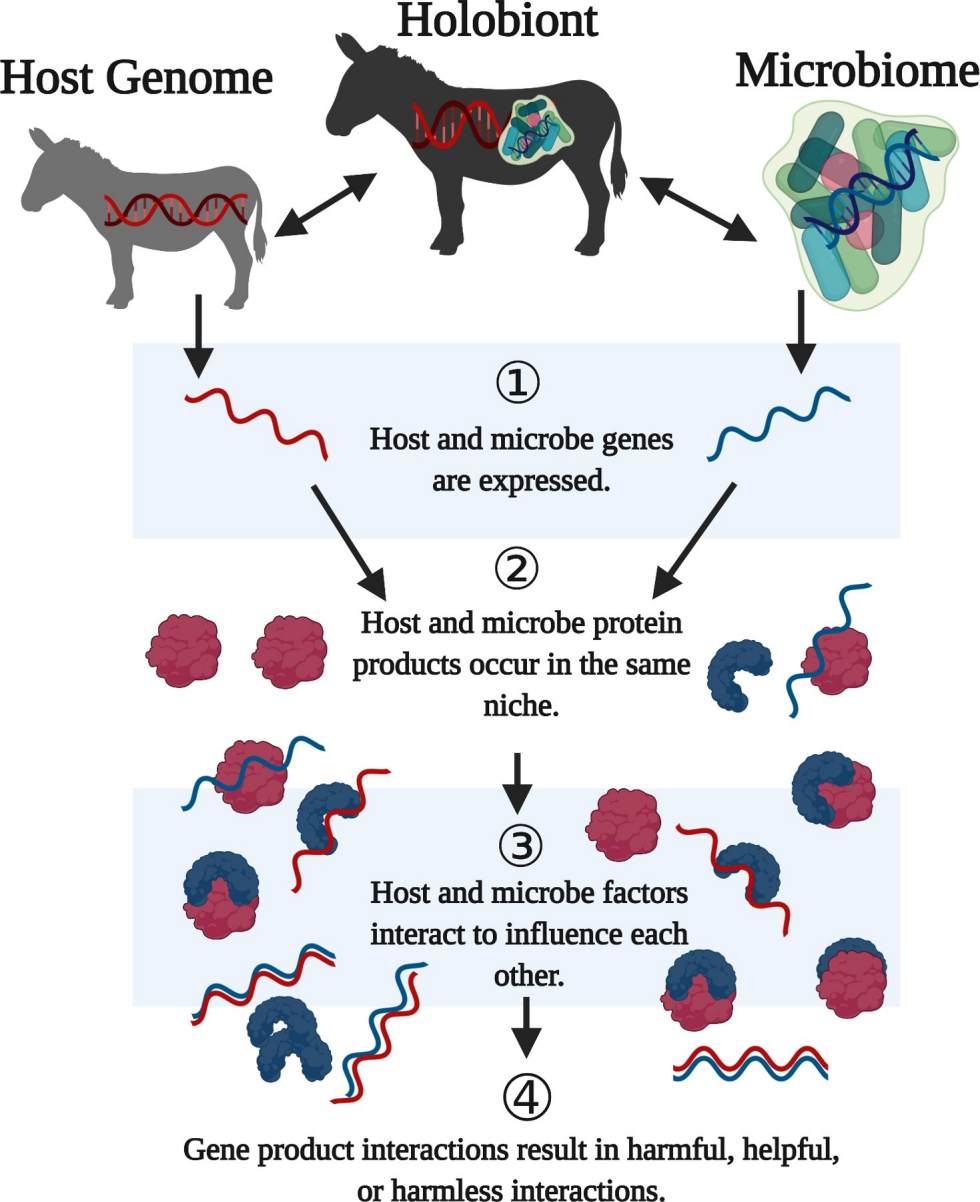
Host of interactions. Diagram depicts the two main parts of the holobiont—the host and associated microbial community. **(1)** Each of these components encode transcripts **(2)** and proteins **(3)** that, in turn, can function alone or together either intragenomically or intergenomically. **(4)** Helpful, harmful, and harmless phenotypes may occur, and the net outcome of these interactions varies with the hologenotype of the host background and presence of other microbes. Created with BioRender.com.

Microbiomes, inclusive of intracellular and extracellular microorganisms, are often distinguishable across host body sites within species [14–20]. Within each site, they can act in a context-dependent manner as harmful, helpful, or harmless to hosts—with potentially integrated metabolisms and interacting gene products **(Fig 1)**. Since the earliest hypotheses of the bacterial nature of mitochondria in eukaryotic cells, microorganisms and their interactions with hosts have been put forth as an engine of novelty that may spur the origin of new species [21]. Microbes can facilitate metabolism, cellular growth, fitness, development, behavior, and competition with other microbes [22–27]. They can also scavenge host nutrients, induce host inflammation, release microbial toxins, or modify host reproduction [28–32], including modification of the gametes that can result in embryonic lethality [32–36]. Such reproductive microorganisms can be relevant to speciation owing to their influences on host gametic integrity and embryonic viability [37,38]. Some microorganisms can also be harmless to their hosts through passive associations such as when microbes thrive on excess nutrients within the host environment [39].

## Species concepts, microbial transmission routes, and phylosymbiosis

Before discussing hybridization cases in animals that cause changes in the microbiome compositions and/or function, it is important to frame the text with a species concept. We adhere to the Biological Species Concept in which species are reproductively isolated groups comprised of potentially interbreeding individuals [40,41]. Hybrids are the result of mating between two species or diverging populations, and the hybrids can suffer from postmating isolation barriers such as sterility and/or inviability [42–47]. The Biological Species Concept was originally formulated for sexually reproducing host species, including plants and animals that are potentially hybridizable in the laboratory. However, the utility of the concept for microorganisms is under active consideration for asexual microorganisms that readily exchange DNA via horizontal gene transfer [48].

**Fig 2:**
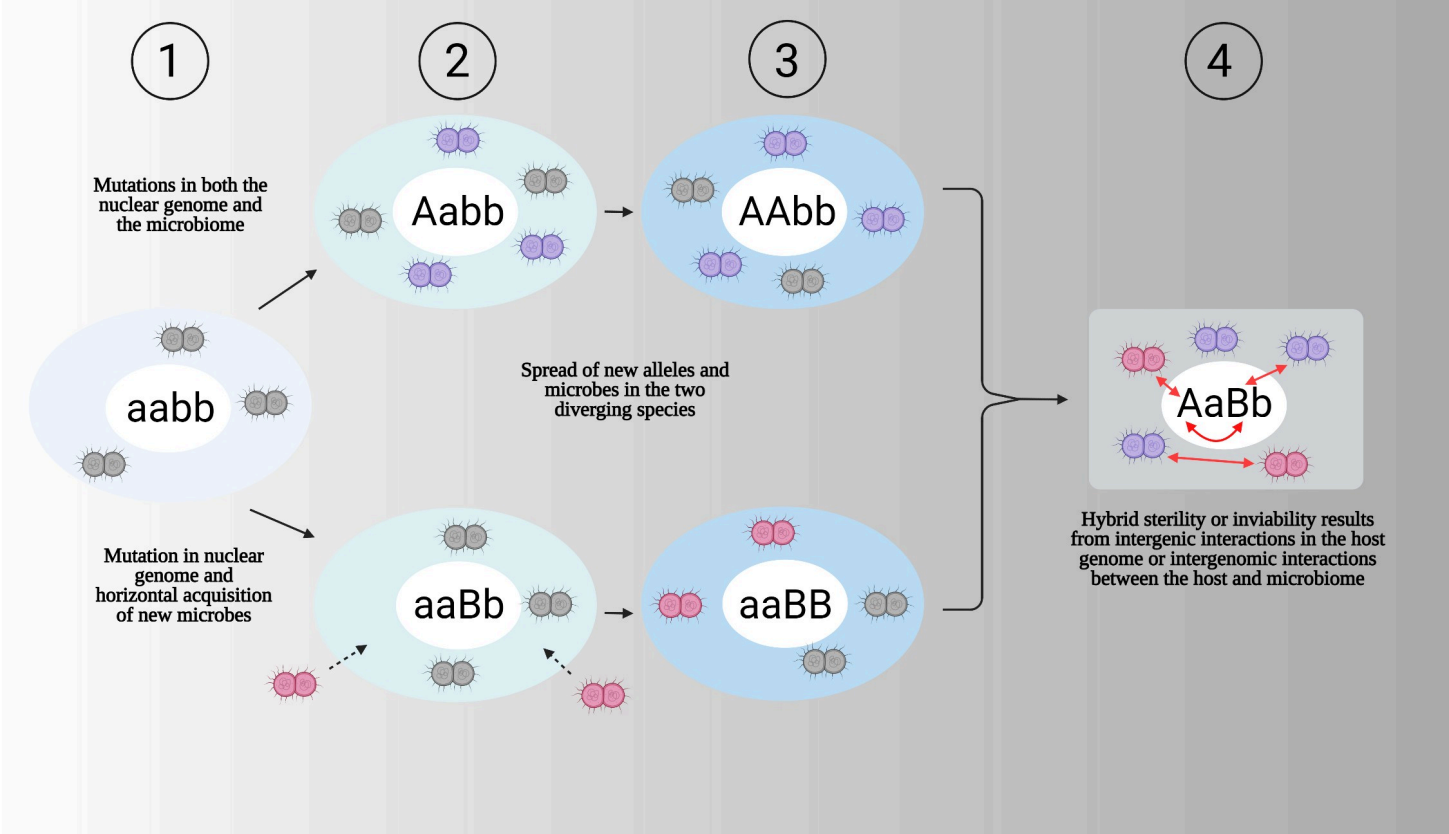
The consequences of changes in the host genome and microbiome on hybrid inferiority. **(1)** The ancestral species nuclear genome (inner white circle) and microbiome (outer blue circle) can change by descent with mutation in both or horizontal transfer and loss of microbes over time. **(2)** After splitting into 2 populations, independent, nuclear mutations accrue in hosts under a standard genetic model of hybrid incompatibility in which separate loci aa and bb mutate and diverge in the 2 populations to AA and BB. Additionally, changes in the microbiome occur due to mutations and horizontal transfer of new microbes. **(3)** The microbiome can be further influenced by loss of microbial members. **(4)** Over evolutionary time, the 2 new holobiont species sufficiently diverged so that they produce sterile or inviable hybrids because of either negative intragenomic (A nuclear locus negatively interacts with B nuclear locus) and intergenomic interactions between different microbes or between members of the microbiome and the host. Created with BioRender.com.

A central question is how reproductive isolation evolves and leads to hybrid maladies such as sterility or inviability. Here, we use the Dobzhansky–Muller model to explain this genetic incompatibility. In the conventional model, hybrid dysfunction between species can arise when two nuclear alleles of ancestral genes aa and bb independently mutate and evolve into derived alleles AA and BB in separate lineages **(Fig 2)** [49]. AA and BB function normally within species, but their gene products in hybrids negatively interact because the derived alleles never evolved together within the same host species, thus resulting in hybrid sterility or inviability [40–47,50]. By extending the Dobzhansky–Muller model to include the host-associated microbiome, one can then ask how do changes in host-associated microbes between the species (for instance, by either mutation or horizontal acquisition of new microbes) impact the number of possible hybrid maladies. Moreover, the impacts of microorganisms on hybrid fitness follow a conceptual continuum to the impacts of macroscopic parasites on hybrid fitness, which has been previously reviewed [28]. We previously demonstrated that a holobiont-based model inclusive of the host and microbiome produces more incompatibilities than a nuclear model alone [38], and these results are in part graphically shown in the schematic below **(Fig 2)**.

As the schematic shows, host species and their hybrids can acquire and cultivate their microbiome through vertical and/or horizontal transmission. Early windows of microbiome exposure and colonization often include the hatching environment, transovarial transmission of endosymbionts, and birth route [33,51–56]. Subsequent windows of microbiome acquisition and colonization across development may include transmission events from diet, socialization, environment, among other factors [57–70] While parental transmission can contribute to long-term fidelity of host–microbiome associations, recurrent associations between hosts and microbes can also be established from environmental sources if, for instance, there is a host selective filter that gardens certain microbes, or vice versa if microbes preferentially colonize and replicate in certain hosts. In other words, vertical transmission is not necessary for holobiont associations to emerge, as environmental acquisition every generation can establish the same taxonomic or functional host–microbe associations anew [62]. Host selection mechanisms (such as the host possessing a gene that influences bacterial titers) are commonly studied [59–62,70].

Without knowing the transmission routes of all members of the microbiome across closely related species, it is notable that the pattern of phylosymbiosis has emerged as a widespread, though not universal, trend in the microbiome sciences. Phylosymbiosis occurs when hosts harbor less microbiome variation within species than between species, and microbiome compositional relationships (i.e., beta diversity) mirror the evolutionary relationships of the host species [61,62,71–74]. As such, more closely related host species harbor more similar microbiomes in parasitoid wasps [75], termites [76], deer [77], mice [73,78], primates [79], and others. As noted in the preceding paragraph, vertical and/or horizontal transmission can establish holobiont compositions underpinning a trend such as phylosymbiosis. Results from interspecific transplants of phylosymbiotic microbiomes between related host species support the hypothesis that selective pressures shape holobiont compositions and phylosymbiosis. For example, fitness (for instance, survival) in *Nasonia* parasitoid wasps and performance (for instance, digestibility) in *Peromyscus* deer mice are reduced in an evolutionary-informed manner upon exposure with increasingly different microbiomes from related host species [73,80]. These results are akin to the costs experienced upon mitochondrial introgressions among related host lineages [81,82] and suggest that natural selection can drive phylosymbiotic changes within parental species that may, in turn, contribute to the evolution of deleterious interactions between hybrids and their microbiomes (**Fig 2**).

## Hybridization and host–microbiome interactions

**Fig 3:**
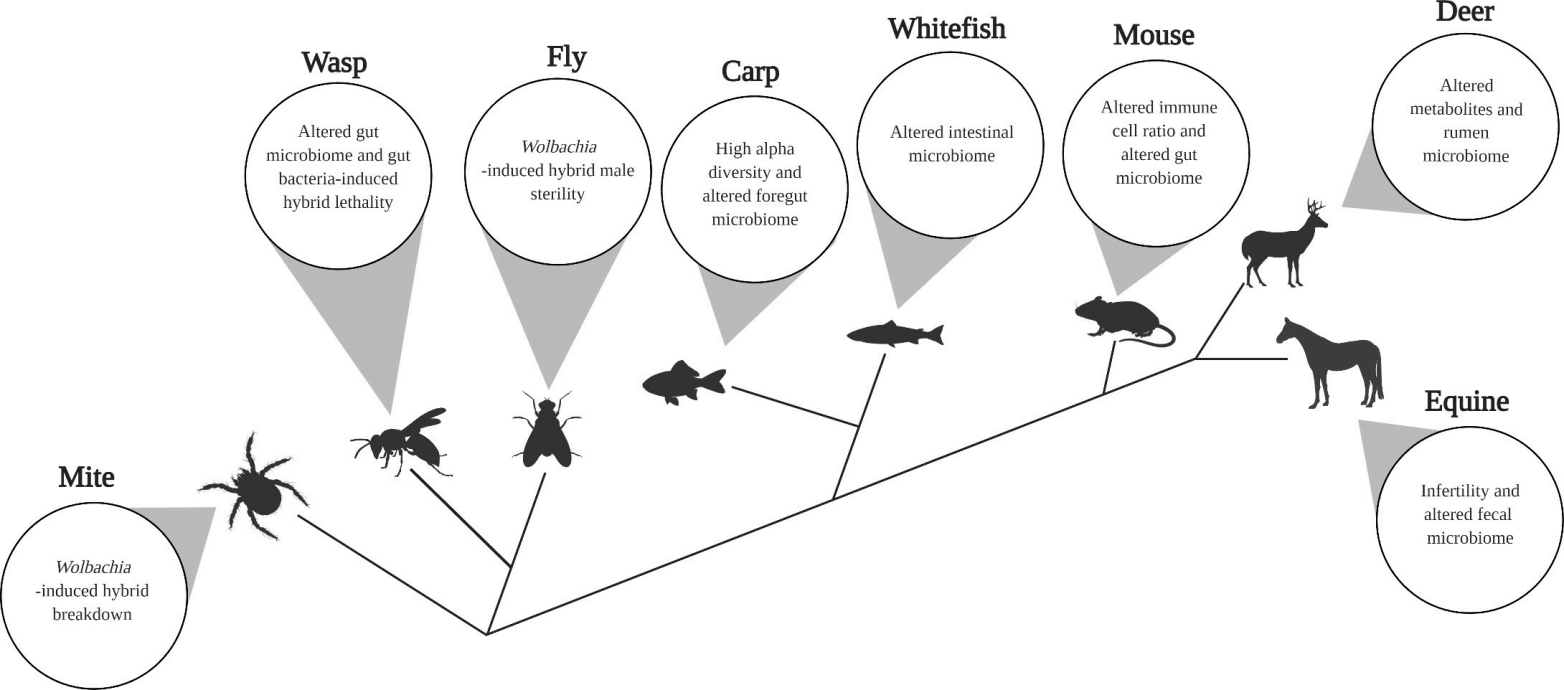
Microbiome alterations occur in diverse hybridizations across animals. Illustrative evolutionary tree depicting examples in which microbial community changes occur in animal hybrids and sometimes impact postmating reproductive isolation. Created with BioRender.com.

In considering the effects of the microbiome on hybridization and speciation on a case-by-case basis **(Fig 3)**, the 8 systems highlighted in **Box 1** likely only scratch the surface of the range of results. As such, understanding the impacts of hybridization on microbiomes will require concerted efforts and exchanges to merge approaches, lexicons, and concepts among the subdisciplines of the life sciences, namely to integrate microbiology more deeply into the origin of macrobial species. Below, we illustrate exemplars of the relationships between hybridization, the microbiome, and microbiome-related metabolites.

**Box 1. Eight case systems offer insight into the relationship between hybridization and the microbiome**

- Mites. In the two-spotted mite *Tetranychus urticae*, *Wolbachia* cause partial cytoplasmic incompatibility (CI) in the F1 generation and hybrid lethality in the F2 males derived from the surviving F1 females in the CI cross [85]. One hypothesis that singularly explains both generations of lethality relates to CI, which causes paternal chromatin defects that kill embryos created between *Wolbachia*-infected males and uninfected females. However, incomplete CI and thus modification of the paternal chromatin could lead to an “aneuploidy hangover” in which surviving F1 females pass on CI-associated aneuploidy to F2 haploid male offspring; and these males subsequently die owing to the aberrant chromosome configuration [85].
- Wasps. The *Nasonia* parasitoid wasp system exemplifies the layered effects of different microorganisms on hybridization. Curable, *Wolbachia*-induced F1 hybrid lethality occurs before the evolution of other pre- or postmating isolation barriers [86,99]. In the F2 hybrid males that are haploid recombinants of the F1 hybrid mothers from the most distantly related species, *Nasonia vitripennis* and *Nasonia giraulti*, the microbiome community changes, wasp larvae undergo hypermelanization, and hyperexpression of the immune system occurs. These catastrophic events associate with most of the hybrid offspring dying during larval development [8,38,87]. Notably, when the *Nasonia* hybrids are reared germ-free, the F2 hybrid male lethality is rescued [87]. Moreover, a member of the wasp microbiome that dominates in hybrids is genomically identical to those that occur in parents, thus supporting a tenet of hologenomic speciation in which resident microbes contribute to hybrid defects following changes in the host genotype [86].
- Flies. The endosymbiont *Wolbachia* causes hybrid male sterility in *Drosophila paulistorum* [84]. In select semispecies hybrid crosses, the sterility can be cured by the administration of antibiotics (toyocamycin nucleoside and gliotoxin) to the parental mother [100]. Homogenates of sterile males that were injected into recipient adult females caused sterility in their male progeny, implicating a maternally derived bacteria (later discovered to be *Wolbachia*) in the induction of male sterility [84,101].
- Carp. In reciprocal F1 hybrids of two invasive species of North American carp, *Hypophthalmichthys nobilis* and *Hypophthalmichthys molitrix*, the bacterial genera *Fusobacteria* and *Firmicutes* were enriched in only one of the hybrid foreguts (female silver carp *H*. *molitrix* x male bighead carp *H*. *nobilis*); there were also intermediate abundances (relative to that of the two parents) of the phyla Cyanobacteria and Bacteroidetes in the hybrid foreguts [92]. The hybrid carp also contained higher gut microbial alpha diversity and an elevated amount of gut microbial genes related to putative cyanophycinase enzymes that may assist digestion of nitrogen/carbon reserve polymers, according to predicted functional capabilities based on the 16S-based taxon data [92].
- Whitefish. In reciprocal crosses of lake whitefish varieties in *Coregonus clupeaformis*, F1 hybrids possess distinguishable microbiomes relative to their parents, and these differences are driven by bacterial genera specific to the two hybrid genotypes [102]. While the whitefish fed on a mixed diet of two food types, some individuals were observed to prefer one food type or both. Within the mixed diet group, a lower abundance of Firmicutes and a higher abundance of Proteobacteria were found in reciprocal hybrids when compared to parental whitefish [102]. The opposite trend of a higher abundance of Firmicutes and a lower abundance of Proteobacteria in hybrid fish was observed when the hybrids consumed only one particular food type. Another study on freshwater lunt snout bream *Megalobrama amblycephala* and carnivorous topmouth culter *Culter alburnus* found that hybrids possessed an intermediate gut length relative to their parental species, and the microbiota composition in hybrids was significantly different from one of the parental species [91].
- Mice. Subspecies of both lab-reared and wild western house mice (*Mus Mus musculus* x *Mus musculus domesticus)* that diverged 0.5 Myr ago can produce hybrid offspring, but they suffer from reduced fertility [103,104] and increased gastrointestinal tapeworm susceptibility. These hybrids also possess distinct gut microbiomes from that of their parental species, and over a dozen genetic loci explain 14.1% of the total variation in microbial community structure in the hybrids. Moreover, microbial abundance variation in hybrids associated with differential expression of immune genes and hybrid gut pathology including ulcerations of the epithelium, accumulation of inflammatory cells, and appearance of organized lymphoid structure [89]. The abundance of immune cells, specifically the ratio of CD4+:CD8+ T cells in intestinal immune tissues, was also different between the parental species, and this ratio in the F2 hybrid mice had greater variance resulting in a pattern that overlapped across both parental mice species [89].
- Deer and elk. The most abundant microorganisms of sika deer and elk rumen include the bacterial phyla Bacteroides and Firmicutes, the archaea *Methanobrevibacter* spp., a protozoan *Entodinium* spp., and the fungi Neocallimastigaceae AL6 and *Cyllamyces*. However, when sika deer (*Cervus nippon*) and elk (*Cervus elaphus*) produce hybrid offspring, the microbiome shifts relative to the parental species, causing an increase in the abundance of Fibrobacter bacterial species or a decrease in the abundance of *Quinella* bacterial species, depending on the direction of the host species cross [77]. In all deer–elk hybrids, the abundance of *Acetitomaculum* bacterial species significantly increased, and various microbial taxonomic changes in the rumen microbiome accompanied shifts in metabolites, especially those involved in carbohydrate, energy, amino acid, and lipid metabolism [77].
- Equine. Hybrids of ponies and donkeys that were all fed on the same diet exhibit significant changes in their fecal bacterial microbiomes and fungal mycobiomes relative to one or both of the two parental species [93]. For example, in hybrids, the fecal mycobiome exhibits markedly less variation than both parental species and is resultantly distinguishable from the range of variation in the parental species. The genus *Piromyces* was generally more common and abundant in hybrids versus parental species. Hybrid fecal bacterial microbiomes were also distinct from donkeys and overlap with ponies [93].

Speciation via endosymbiosis is perhaps the most well-appreciated exemplar of microbe-assisted reproductive isolation in hybrids [83]. For instance, in the *Drosophila paulistorum* fruit fly of Central and South America, several semispecies are reproductively isolated in agreement with Haldane’s rule (the heterogametic sex is sterile or inviable). In particular, *D*. *paulistorum* hybrid males are sterile due to the testes overproliferation of *Wolbachia*, a widespread bacterium of the reproductive system of arthropods [84]. *Wolbachia*’s symbiotic presence in the germline may result in host selective pressures to cope with the bacterial symbiont, leading to host accommodation of the bacteria within species and breakdown of that accommodation in compromised hybrids. Some *Wolbachia–*host combinations intriguingly cause both hybrid lethality in the F1 generation and hybrid breakdown in the F2 generation. For instance, in *Tetranychus urticae* mites, the *Wolbachia* not only cause cytoplasmic incompatibility (CI) and thus F1 hybrid reductions, but they also cause F2 hybrid lethality due to an “aneuploidy hangover” in which surviving F1 hybrid females pass on CI-associated aneuploidy to F2 haploid male offspring; these hybrid males die owing to aberrant chromosome configuration [85]. Since CI effects are rarely measured past the F1 generation, the frequency of F2 symbiont-associated breakdown is currently unknown and likely more important than currently appreciated.

In the genus *Nasonia*, which harbors 4 parasitoid wasp species that diverged between 0.2 and 1.0 Myr ago depending upon the species pair, there are multiple layers of hybridization impacted by symbiotic bacteria. First, different CI *Wolbachia* in each of the parental wasp species cause strong F1 hybrid lethality, and CI-induced reproductive isolation can evolve early in the speciation process [86]. Second, once cured of their *Wolbachia* in the lab, F1 hybrids readily survive and produce F2 hybrids that then succumb to approximately 90% hybrid lethality during advanced stages of larval development [87]. Reexposing F2 germ-free hybrids with members of their parental-associated bacteria leads to higher death rates compared to parental species controls, thus emphasizing that interactions in hybrids between the microbiome and recombinant host genome cause the F2 hybrid lethality [87]. Selective pressures on the hologenome likely contribute to the F2 hybrid breakdown as microbiome transplants between wasp species lead to reduced wasp fitness in an evolutionary-informed manner [80]. Instances of microbiome-dependent hybrid lethality coupled with evidence of selective pressures on phylosymbiosis within species are important for contextualizing why hologenomic reproductive isolation evolves within species.

Given the Large Immune Effect on hybrid incompatibilities [38], and the hypothesis that the vertebrate adaptive immune system evolved to manage more complex microbial communities [88], vertebrates are an important group to investigate interactions that contribute to reproductive isolation and speciation. For example, mice hybrids between *Mus musculus* and *Mus domesticus* exhibit increased gut pathology in association with both a compromised immune system and an altered gut microbiome [89]. In sika deer and elk, as well as their hybrids, the rumen microbiome facilitates the absorption and metabolism of nutrients [77]. When parentals are compared to their hybrids, several taxonomic changes occur in their microbiomes. For example, the presence of *Acetitomaculum* bacteria, which may be associated with the conversion of lactate to acetate, is notably higher in the hybrids [77,90]. These findings suggest that holobiont hybrids may not metabolize in the same way as nonhybrids due to the breakdown in the microbiome and host–microbiome metabolic crosstalk. In whitefish, specific members of the *Coregonus clupeaformis* microbiome shift in hybrids relative to parents, suggesting a breakdown in selection on fish–microbiome interactions within species [91]. Similarly, hybridization of two invasive species of North American carp, *Hypophthalmichthys nobilis* and *Hypophthalmichthys molitrix*, can cause significant differences in microbiome composition and functional potential [92]. Finally, hybrids of ponies and donkeys display a markedly different microbiome than their parentals [93]. The functional implications of these changes remain to be investigated, but impacts on fiber digestibility, nutrient intake, and gut disease are candidates for phenotypic consequences in hybrids.

## Lessons learned from case studies

This brief synthesis of empirical evidence highlights that microbiomes are altered in hybrids across animal diversity, and in some cases they directly cause reproductive isolation. Although the sample of exemplars are not exhaustive, they are not limited to one type of microbe, host system, or isolation mechanism. Rather, they span specific endosymbionts such as *Wolbachia* and diverse microbial communities across the animal phylogeny, impact various stages of animal development and anatomy, and cause reproductive isolation in the F1 and F2 generation. Thus, we conclude that hybridization impacts on the microbiome and vice versa will prove general, and that conclusion raises the stakes on a topic that is arguably among the most understudied in hybridization and speciation. We do not suggest a necessity to rethink the whole field, but rather to acknowledge that a eukaryocentric view has dominated animal and plant speciation studies for the past century. Recent work suggests that the evolution of hybrid maladies arises from host accommodation of long-term endosymbionts, selfish drives of symbionts, and selection on holobiont compositions that break down with costs on fitness and performance. We must also emphasize that these hybridization exemplars are not encompassing of plants, and there are instances of microbiome-mediated impacts on premating isolation and host adaptation that we and others have previously discussed [94–96].

## Concluding remarks

In this perspective, we call attention to literature on the microbiology of animal hybrid hosts to equip evolutionary biologists, developmental biologists, biochemists, and microbiologists with case examples of how the microbiome sheds important light on speciation and hybridization. We set out to answer the following questions: How does hybridization affect the microbiome? Can hybrid maladies be facilitated by changes in the microbiome? And how do hybrid organisms differ from their parentals? The exemplars presented here offer a steppingstone to studies that comprehensively interrogate (i) the distinguishable nature of microbiomes across host species; (ii) the characterization and causes of microbial differences between hybrids and their parental species; and (iii) the microbial and host components of animal reproductive isolation and speciation.

With the advent of high-throughput, holo-omic technologies for nucleotide and protein sequencing and metabolomics, there is an emerging need for integratively studying reproductive isolation and speciation through various scales of taxonomy (bacteria, fungi, viruses, archaea), anatomy (gut, reproductive tissue, etc.), and host diversity (invertebrates, vertebrates). Moving forward, there are still several questions outstanding including: What types of hybrid reproductive isolation are most frequently impacted by microorganisms? Does natural selection in parental holobionts often lead to divergence in the host genome or microbiome and ensuing hybrid maladies? How have well-characterized systems of hybrid maladies overlooked the influences of microbial communities that previously went unmeasured in experiments? How do bacteriophages and viruses impact microbial community changes in hybrids?

More studies, similar to those reviewed here, are necessary to fully appreciate the interplay between the microbiome and hybridization. Over the past 2 decades, biology has become invigorated by what evolutionary microbiologist Carl Woese designated as the “sleeping giant” of biology—the microbial world [97]. Awoken and now conventionally studied, investigations of the microbiome offer increasing relevance to diverse subfields of biology, yet speciation in various macrobial systems has lagged behind other fields that have intensively interrogated and integrated the microbial world. To exclude the host-associated microbiome in experiments, concepts, and theory is to exclude vital parts of the biological system of a holobiont. We conclude with a suggestion and call to action that studying hybrid microbiomes is likely to be one of the most fruitful areas of future speciation research based in part on the case systems outlined above. As Carl Woese also wrote, “Biologists now need to reformulate their view of evolution to study it in complex dynamic-systems terms” [98]. This call does not translate to a radical change in paradigm, but rather embraces robust integration across biological hierarchies, concepts, approaches, conferences, and teams.
